# Research on machine learning-based clinical prediction models: a bibliometric analysis

**DOI:** 10.3389/fonc.2026.1786176

**Published:** 2026-04-01

**Authors:** Qinshan Li, Mingli Zeng, Danmei Liang, Xuemei Zheng, Xiaoxia Zhang, Ruben Martin-Payo

**Affiliations:** 1 West China School of Nursing, Sichuan University, Chengdu, China; 2 Cancer Center, Division of Head and Neck Tumor Multimodality Treatment, West China Hospital, Sichuan University, Chengdu, China; 3 Cancer Center, West China Hospital, Sichuan University, Chengdu, China; 4 Cancer Center, Shangjin Nanfu Hospital, West China Hospital, Chengdu, China; 5 Facultad de Medicina y Ciencias de La Salud, Universidad de Oviedo, Oviedo, Spain; 6 Precam Research Group, Instituto de Investigación Sanitaria del Principado de Asturias (ISPA), Oviedo, Spain

**Keywords:** bibliometric analysis, CiteSpace, clinical prediction models, machine learning, VOSviewer

## Abstract

**Background:**

Machine learning (ML) has emerged as a transformative approach for developing high-performance clinical prediction models (CPMs). By leveraging multidimensional patient data, ML enables more accurate disease risk stratification, prognostic assessment, and clinical decision-making. In recent years, research on CPMs has expanded rapidly, with nearly 250,000 publications indexed as of 2024. Despite this remarkable growth, a comprehensive bibliometric analysis of the field is currently lacking.

**Objective:**

This study aimed to analyze the global research status, evolutionary trends, and thematic hotspots of machine learning-based clinical prediction models (ML-CPMs) through bibliometric and visualization techniques.

**Methods:**

Publications related to ML-CPMs were retrieved from the Web of Science Core Collection and the Scopus database (up to May 9, 2025). Bibliometric analyses were performed using various tools, including R, VOSviewer, and CiteSpace, to generate annual publication trends, collaboration networks, and journal distributions, as well as co-citation, clustering, and keyword analyses.

**Results:**

A total of 8,619 publications (8,000 original articles and 619 reviews) from 118 countries were identified. Since 2015, annual publications have grown exponentially (*R²* = 0.9919). While China led in total publication volume, the United States maintained the highest academic influence (H-index = 105; Total Citations = 66,788). Harvard University and *BMC Medical Informatics and Decision Making* emerged as the most productive institution and journal, respectively. Tian J from the Chinese Academy of Sciences led in publication count, while Wynants L from KU Leuven in Belgium recorded the highest citation frequency. Key research hotspots include algorithm optimization, multimodal data integration, and model interpretability, with clinical applications primarily focused on oncology, cardiovascular diseases, and critical care medicine.

**Conclusion:**

Research on ML-CPMs has experienced rapid global growth over the past decade, forming extensive international collaboration networks. However, challenges such as limited interpretability, data heterogeneity, and privacy concerns persist. Future studies should prioritize external validation, clinical applicability, and the integration of human-AI collaborative decision-making to ensure robust implementation in real-world clinical settings.

## 1 Introduction

Clinical Prediction Models (CPMs) quantify disease risk or prognosis by integrating multidimensional patient data and serve as essential tools for supporting clinical decision-making and optimizing resource allocation, thereby improving healthcare quality and efficiency (1). In recent years, machine learning (ML), a widely adopted branch of artificial intelligence (AI), has become a key approach for developing high-performance predictive models (2). ML can automatically extract complex features and capture nonlinear relationships, demonstrating remarkable advantages in various medical domains such as image analysis, electronic health record (EHR) mining, and multi-omics data integration (3–5). ML-based clinical prediction models (ML-CPMs) have not only facilitated the clinical translation of predictive modeling in oncology, cardiovascular medicine, and critical care but have also shown great potential in prognostic assessment, risk stratification, and early warning applications (6–8).

Recent estimates suggest that by 2024, the total number of publications related to CPMs will have reached approximately 250,000 (9), reflecting the substantial research volume in this field. Given the rapid growth of the literature, several bibliometric analyses have been conducted focusing on specific disease areas. For instance, research on cervical cancer has shown that risk prediction models commonly employ random forest and support vector machine algorithms, yet they often lack external validation and remain limited in interpretability (10). In the intensive care field, research hotspots have primarily centered on early warning of clinical deterioration, mortality risk prediction, and patient phenotyping, with machine learning and deep learning approaches being the dominant methodologies (11). Studies in diabetes have reported a continuous increase in publication volume, with research themes mainly focused on diabetic retinopathy and diabetic foot, predominantly utilizing machine learning and deep learning techniques (12, 13). At a broader disciplinary scale, bibliometric research has also addressed AI and ML in healthcare as comprehensive technological domains, encompassing diverse applications such as medical imaging, clinical decision support, disease diagnosis, and risk prediction, and highlighting the expanding role of data-driven technologies in medicine (14–16). However, ML-driven CPMs have not been examined as a distinct research domain in the bibliometric literature.

Bibliometric analysis, which applies statistical methods to quantitatively evaluate academic publications, serves as a valuable tool for revealing research trends, collaboration networks, and scholarly influence within a field (17). Therefore, this study aimed to integrate statistical and visualization techniques to characterize the research landscape, developmental trends, and emerging hotspots in the field of ML-CPMs.

## 2 Materials and methods

### 2.1 Data sources and search strategies

Web of Science Core Collection (WoSCC) and Scopus were selected because they offer broad multidisciplinary coverage and provide rich citation information (18). Compared with PubMed, which is primarily designed for biomedical indexing and lacks complete citation network data, WoSCC and Scopus are more suitable for bibliometric mapping and citation analysis. On May 9, 2025, literature retrieval was conducted independently in WoSCC and Scopus. A topic-based search was performed using keyword combinations such as “machine learning,” “artificial intelligence,” “predictive models,” and “diagnostic models.” To ensure thematic relevance to the medical domain, subject-based filters were applied at the database level in both WoSCC and Scopus. The complete search strategy is provided in **Supplementary Table 1**.

### 2.2 Literature screening and eligibility criteria

Two researchers independently screened titles and abstracts, with duplicate and non-eligible records removed through manual verification according to the predefined inclusion and exclusion criteria. In cases of disagreement regarding study selection, data extraction, or quality assessment, a third researcher was consulted to resolve discrepancies through discussion and consensus.

Inclusion criteria: (1) development of clinical prediction models using machine learning methods; (2) human subjects; (3) original articles or reviews; (4) written in English; and (5) published from database inception to May 9, 2025. Exclusion criteria: (1) animal or basic laboratory experiments; (2) letters, comments, replies, or corrections; (3) editorials or commentaries; (4) retracted publications; and (5) news reports.

### 2.3 Data processing

Bibliographic records from WoSCC were exported in BibTeX format with full records and cited references, while records from Scopus were exported in CSV format with the full available metadata to improve compatibility for multi-database integration (19). The two datasets were then imported into R 4.5.0 and processed using the bibliometrix 5.0.1 package, which converts records from different sources into a common data structure. Records were subsequently cleaned, standardized, and merged into a unified dataset for downstream analyses across multiple bibliometric tools.

Prior to data analysis, manual normalization was performed for keywords, author names, and country names, including synonym merging, spelling correction, and abbreviation standardization, to ensure consistency and accuracy across different software tools. For instance, “People’s Republic of China,” “Taiwan,” and “Hong Kong” were standardized as “China,” while “Northern Ireland,” “Wales,” “England,” and “Scotland” were grouped under “United Kingdom”.

### 2.4 Data analysis

VOSviewer 1.6.20 was used to analyze and visualize international collaboration among countries, institutions, and authors, as well as journal co-occurrence and keyword co-occurrence networks. In VOSviewer, inclusion thresholds were tailored to each analytical task to maintain interpretability while preserving informative network structure. CiteSpace 6.4.R1 was applied to generate dual-map overlays of journals, keyword timeline views, burst detection maps, and co-citation networks, thereby revealing the evolutionary pathways and emerging frontiers of research themes. For CiteSpace analyses, the time span was set to 1994–2025 with one-year slices and g-index node selection. Pruning strategies were selected according to the analytical task: Pathfinder was used for cluster visualization when appropriate, whereas no pruning was applied to co-citation networks used for burst detection. In addition, Microsoft Excel 2019 and ArcMap 10.8 were employed for data processing and visualization.

## 3 Result

### 3.1 Overall analysis

**Figure 1:**
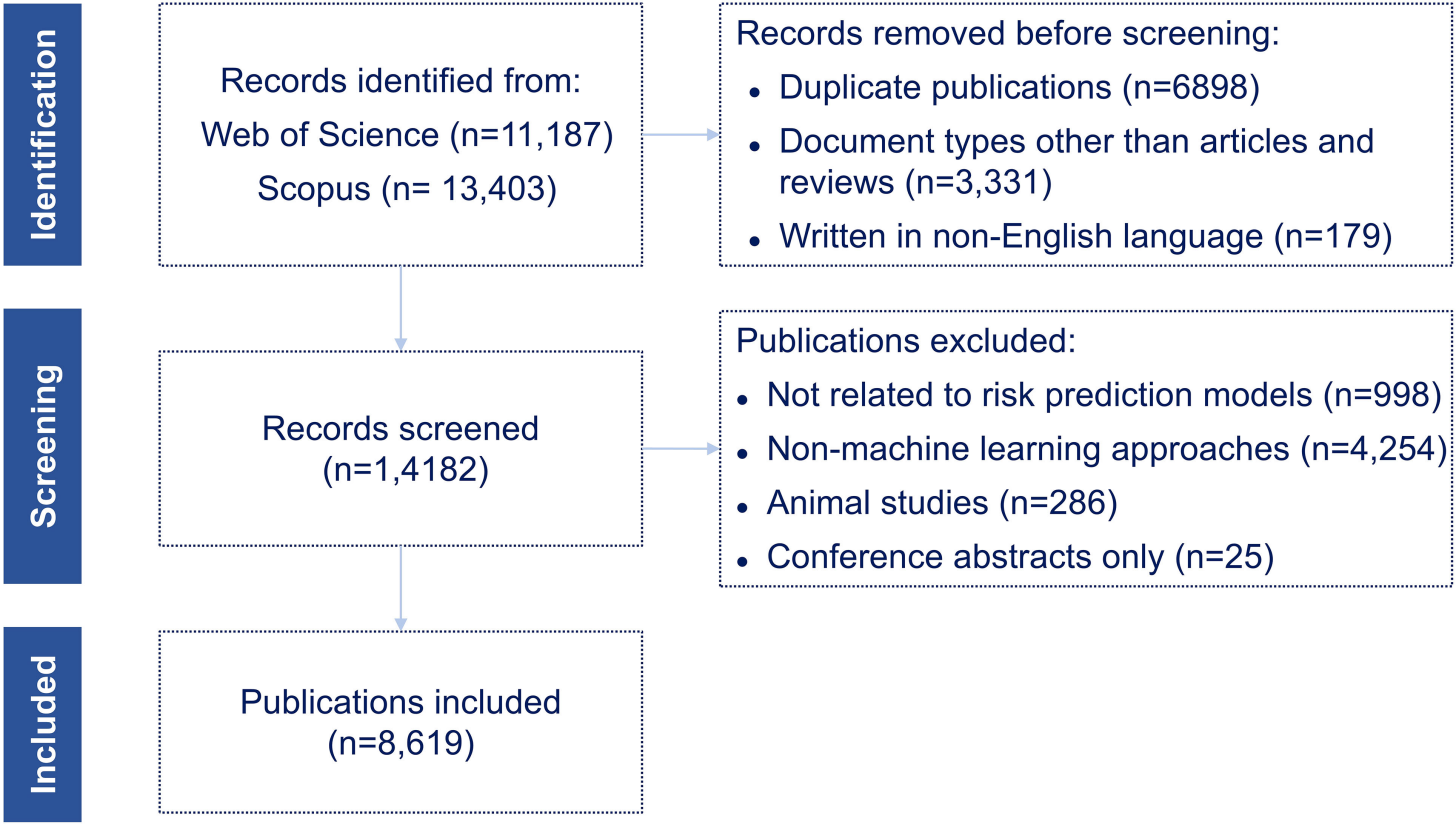
Flowchart of the literature screening process.

A total of 11,187 records were initially retrieved from WoSCC, and 13,403 records were retrieved from Scopus. After merging records from the two databases, removing duplicates, and applying the inclusion criteria, 8,619 eligible publications were retained for analysis, comprising 8,000 original research articles (92.8%) and 619 review articles (7.2%). **Figure 1** illustrates the detailed flowchart of the literature screening process.

### 3.2 Annual publication trends

**Figure 2:**
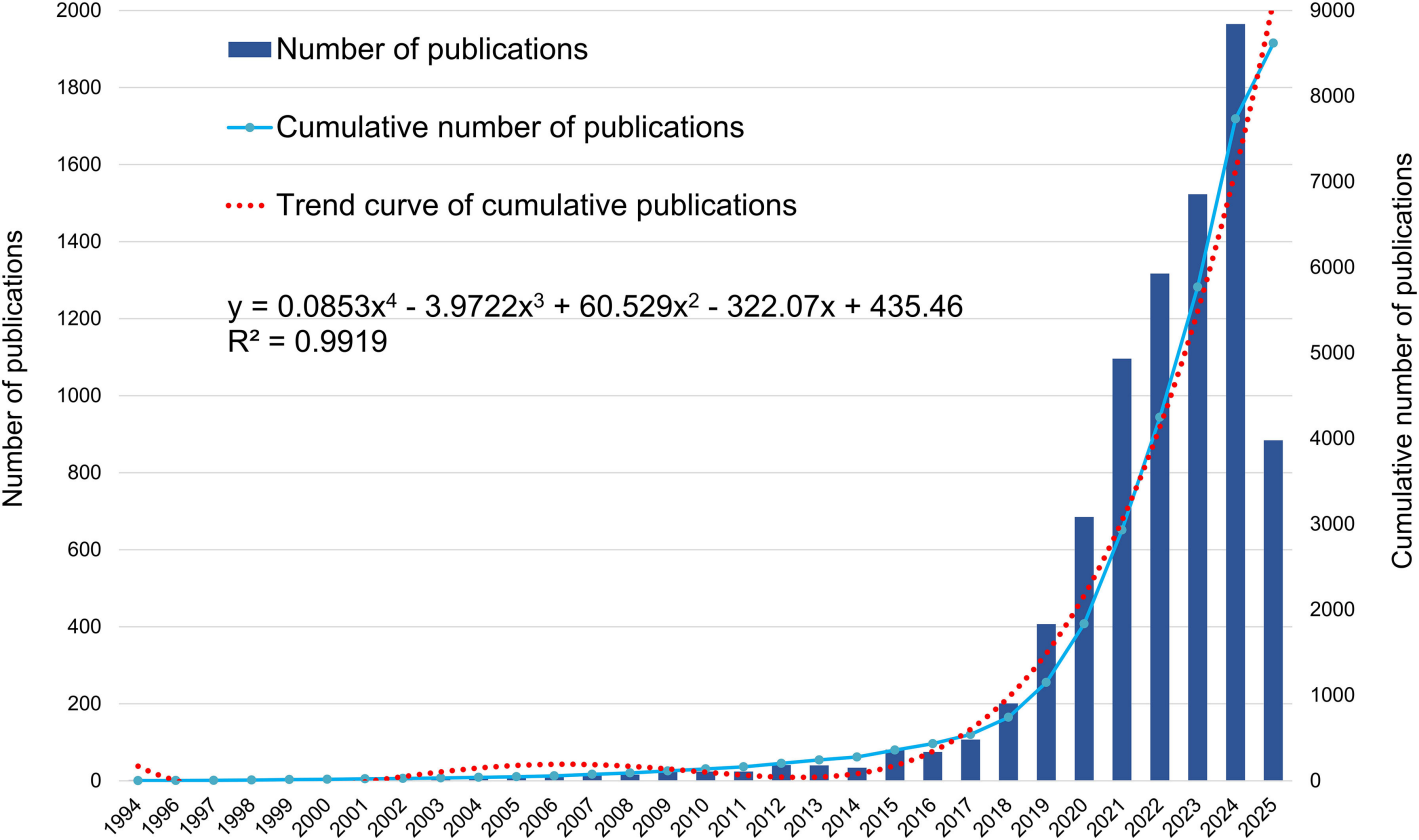
Annual and cumulative number of publications.

The analysis revealed that as early as 1994, studies had attempted to apply machine learning methods to clinical prediction modeling. Woolery LK and Lowell WE independently developed neural network-based models for predicting preterm birth risk and hospitalization duration in patients with mental illness, respectively (20, 21). These studies marked the initial exploration of machine learning in clinical risk prediction. **Figure 2** presents the annual and cumulative publication trends in the ML-CPMs field from 1994 to 2025. Over the past three decades, there has been significant growth, reaching a total of 8,619 by May 2025. Polynomial trend fitting demonstrated a strong correlation between publication volume and time (*R²* = 0.9919), indicating a statistically significant upward trajectory in publication growth.

Further analysis of the annual publication trends suggests that research on ML-CPMs has evolved through four distinct developmental phases. The initial phase, from 1994 to 2006 represented the exploratory stage, with only 1–10 publications per year. The second phase (2007–2014) was characterized by slow growth, peaking in 2012 before a slight decline over the following two years. The third phase (2015–2020) marked a period of accelerated development, during which annual publications increased sharply from 81 to 685. The most recent phase (2021–2025) reflects a period of high research activity, reaching a peak of 1,965 publications in 2024 and projected to surpass 2,000 by 2025.

### 3.3 Analysis of countries

**Figure 3:**
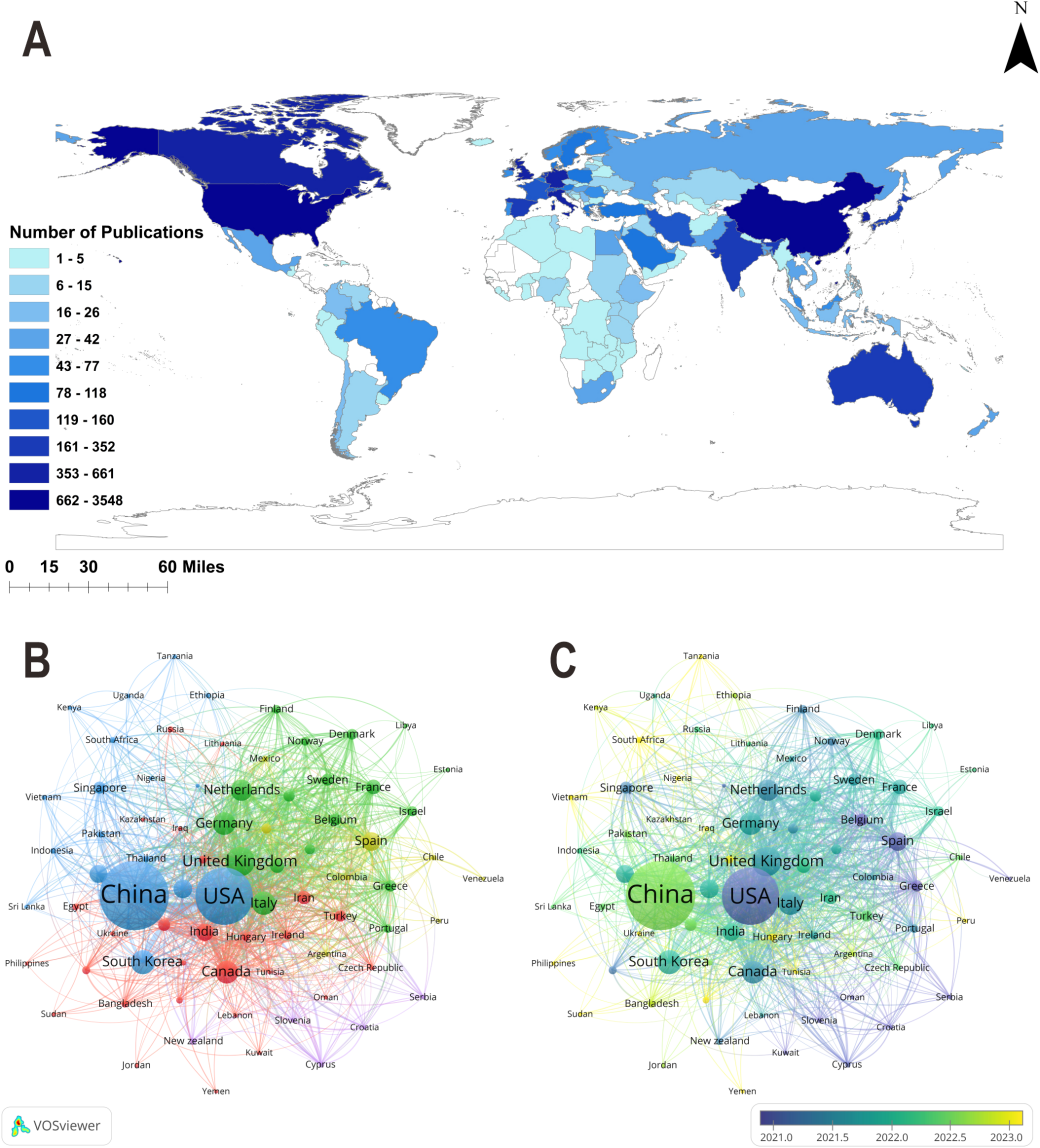
**(A)** Global distribution of publications by country. Darker colors indicate a higher number of publications. **(B)** Collaboration network among countries with ≥5 publications. Node size represents publication volume, and connecting lines denote collaborative relationships between countries. Different colors indicate distinct collaboration clusters. Total collaboration strength is quantified by total link strength, with higher values reflecting closer international cooperation. **(C)** Temporal evolution of international collaboration. Node colors indicate the average publication year for each country, illustrating the temporal distribution of research activity from 2021 to 2023.

A total of 118 countries have contributed to research on ML-CPMs. As shown in **Figure 3A**, China (n = 3,548), the United States (n = 2,423), and the United Kingdom (n = 661) ranked as the top three countries in publication output. Collaboration network analysis (**Figure 3B**) revealed that the United States (TLS = 2,003), the United Kingdom (TLS = 1,195), and China (TLS = 942) occupied central positions within the global cooperation network. Temporal evolution analysis reveals that countries such as the United States, the United Kingdom, Germany, the Netherlands, and Canada had earlier average publication years (around 2021), representing the core research forces in this field. In subsequent years, research activity gradually expanded to emerging regions across Asia and the Middle East, with countries such as China, India, Saudi Arabia, Iran, Turkey, Thailand, and South Africa showing more recent average publication years (after 2022), reflecting an increasing trend toward geographical diversification in global research collaboration (**Figure 3C**).

The publication performance of the top ten countries is summarized in **Table 1**. China ranked first with 3,548 publications (41.2%), although it placed second in total citations (43,208) and H-index (77). The United States, despite a slightly lower publication count, achieved the highest total citations (66,788) and H-index (105), reflecting its greater academic influence in this field. The United Kingdom ranked third with 661 papers (7.7%) and an average of 33.86 citations per article. Notably, the Netherlands, though contributing 352 papers (4.1%), recorded the highest average citation rate (42.60), indicating the high impact of its research output.

**Table 1:** Top 10 productive countries ranked by numbers of publications.

Rank	Country	NP(%)	NC	AC	TLS	H-index
1	China	3,548(41.2%)	43,208	12.18	942	77
2	USA	2,423(28.1%)	66,788	27.58	2,003	105
3	United Kingdom	661(7.7%)	22,381	33.86	1,195	63
4	South Korea	448(5.2%)	6,123	13.67	233	38
5	Italy	410(4.8%)	8,741	21.32	691	44
6	Canada	387(4.5%)	9,848	25.45	643	44
7	Germany	379(4.4%)	10,145	26.77	706	46
8	Netherlands	352(4.1%)	15,037	42.60	706	57
9	Australia	263(3.1%)	7,449	28.32	461	38
10	Spain	259(3.0%)	4,724	18.24	435	38

NP, Number of Publications. NC, Number of Citations. AC, Average Citations. TLS, Total Link Strength. USA, The United States of America.

### 3.4 Analysis of institutions

**Figure 4:**
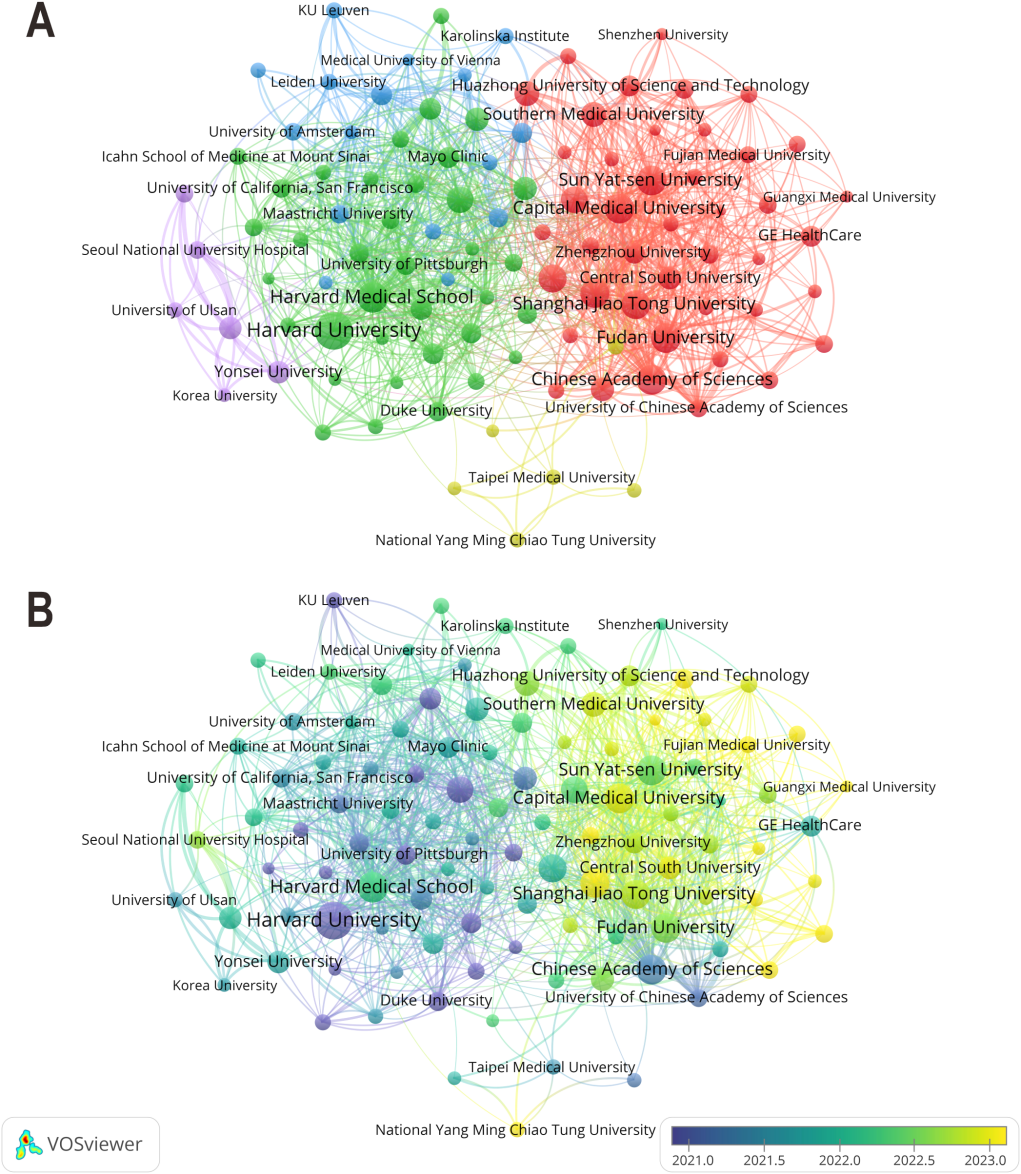
**(A)** Collaboration network of the top 100 research institutions, each contributing at least 19 publications. Each node represents a research institution, with node size indicating publication volume and line thickness reflecting collaboration strength. Different colors denote clusters of closely collaborating institutions. **(B)** Temporal evolution of institutional collaboration. Node colors indicate the average publication year for each institution, illustrating the temporal distribution of research activity from 2021 to 2023.

A total of 9,903 institutions have contributed to publications in the ML-CPMs field. As shown in **Figure 4A**, Harvard University (TLS = 730), Harvard Medical School (TLS = 330), Sun Yat-sen University (TLS = 237), Capital Medical University (TLS = 237), and Fudan University (TLS = 185) hold central positions in the institutional collaboration network. This indicates a high level of research connectivity and cooperative activity. Temporally, as depicted in **Figure 4B**, institutions such as Harvard University, Stanford University, and the University of Toronto were prominent in research before 2021. In contrast, after 2022, Chinese universities including Fudan University, Shanghai Jiao Tong University, and Sun Yat-sen University significantly increased their publication output, reflecting the growing research momentum in Asia.

The top ten most productive institutions are listed in **Table 2**. Harvard University ranked first, with the largest number of publications (n = 312) and the highest H-index (49). The top four institutions were all located in the United States, which highlights the strong academic influence of American research organizations in this field. Institutions ranked fifth and below were predominantly from China, including Sun Yat-sen University, Capital Medical University, and Fudan University. Although Chinese institutions have shown rapid growth in publication output, their average citation counts and H-index values remain generally lower than those of their US counterparts, indicating that there is still room to strengthen their international research impact.

**Table 2:** Top 10 productive institutions with publications.

Rank	Institution	Country	NP	NC	AC	H-index
1	Harvard University	USA	312	12,868	41.24	49
2	Harvard University Medical Affiliates	USA	249	9,800	39.36	45
3	University of California System	USA	223	6,844	30.69	42
4	Harvard Medical School	USA	202	8,217	40.68	39
5	Sun Yat-sen University	China	196	3,614	18.44	31
6	Capital Medical University	China	191	2,101	11.00	24
7	Fudan University	China	190	3,145	16.55	29
8	Chinese Academy of Sciences	China	189	3,948	20.89	36
9	Chinese Academy of Medical Sciences Peking Union Medical College	China	184	2,433	13.22	28
10	Shanghai Jiao Tong University	China	183	2,254	12.32	22

NP, Number of Publications. NC, Number of Citations. AC, Average Citations.

### 3.5 Journal productivity and citation patterns

**Figure 5:**
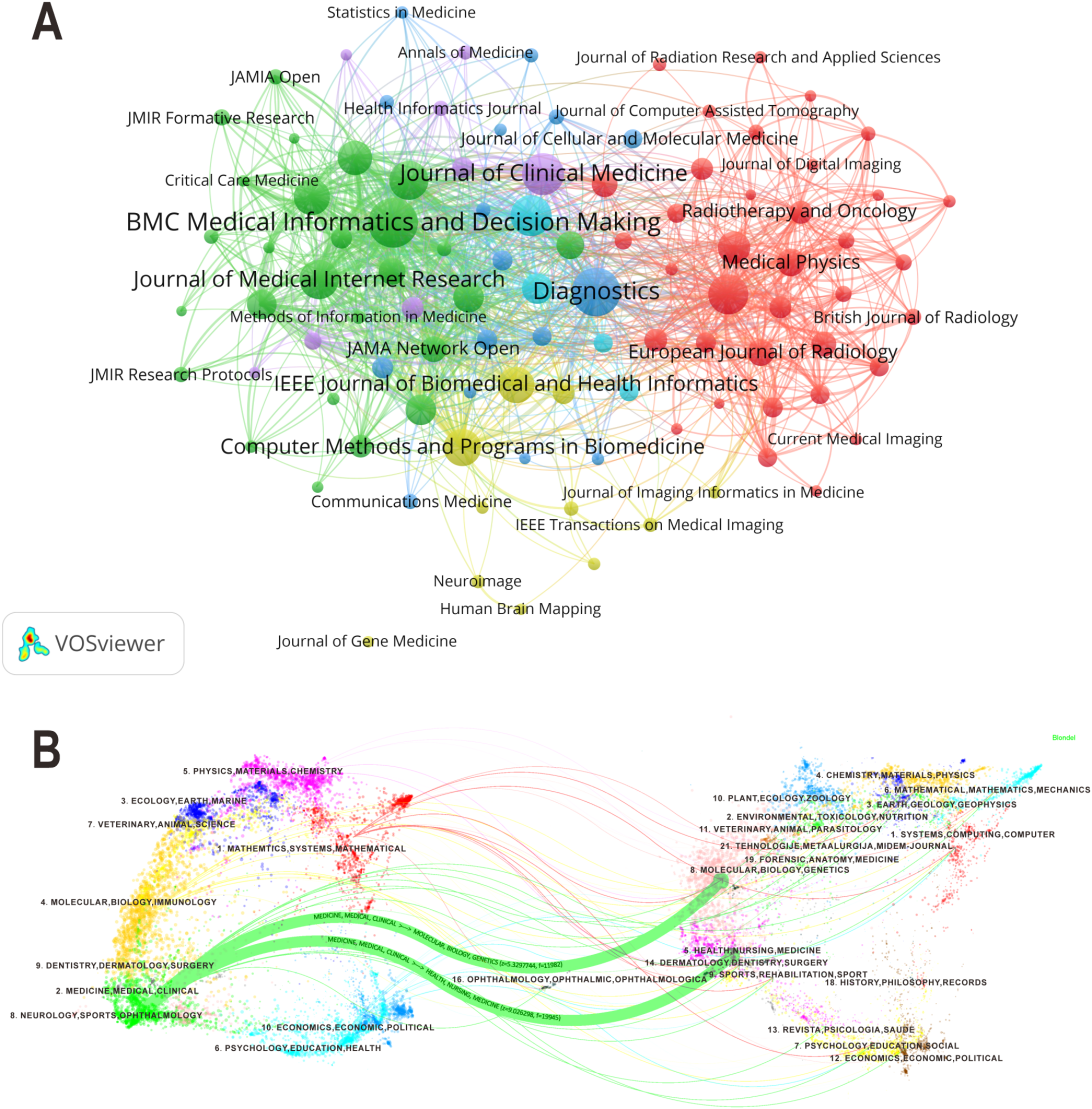
**(A)** Journal collaboration network. Colors denote different clusters, node size indicates publication volume, and line thickness reflects collaboration strength. **(B)** Dual-map overlay of journals. The left side represents citing journals, and the right side represents cited journals. Colored paths illustrate citation relationships across disciplines, where f denotes citation frequency, indicating the intensity of interdisciplinary knowledge flow, and z represents standardized significance, indicating the prominence of each citation path.

The bibliometric analysis identified 677 journals contributing to research on ML-CPMs. The journal collaboration network formed six major clusters, reflecting the field’s diverse research directions (**Figure 5A**). These clusters are medical imaging and radiotherapy (red cluster), medical informatics and digital health (green cluster), brain imaging and neuroscience (yellow), interdisciplinary health informatics (purple cluster), biomedical engineering and computational modeling (light blue cluster), and comprehensive clinical research (dark blue cluster). **Figure 5B** illustrates citation relationships between journals and cocited journals. Two prominent green citation paths were apparent, showing that clinical medicine journals frequently cited nursing and public health (*f* = 19,945, *z* = 5.06) and molecular biology and genetics (*f* = 19,882, *z* = 5.33).

**Table 3** lists the top ten journals by publication volume, which together contributed 2,269 papers, accounting for 26.3% of the total output. Among them, *BMC Medical Informatics and Decision Making* (n = 328), *Diagnostics* (n = 317), and *Journal of Clinical Medicine* (n = 239) ranked among the top three. Six of the ten journals were classified in JCR Q1, with impact factors ranging from 2.9 to 6.8. *IEEE Journal of Biomedical and Health Informatics* had the highest impact factor (6.8), whereas *European Radiology* achieved the highest citation count (6,289 citations, H-index = 43).

**Table 3:** Top 10 productive journals with publications.

Rank	Journal	Country	NP	NC	JCR Quartile	IF (2025)	H-index
1	BMC Med Inform Decis Mak	United Kingdom	328	5,532	Q2	3.8	32
2	Diagnostics	Switzerland	317	2,508	Q2	3.3	23
3	J Clin Med	Switzerland	239	2,420	Q1	2.9	25
4	Front Med	Switzerland	222	1,645	Q1	3	21
5	Eur Radiol	Germany	213	6,289	Q1	4.7	43
6	Int J Med Inform	Netherlands	209	3,966	Q1	4.1	34
7	J Med Internet Res	Canada	202	3,804	Q1	6	31
8	Comput Methods Programs Biomed	Ireland	185	3,651	Q1	4.8	33
9	JMIR Med Inform	Canada	178	2,371	Q2	3.8	23
10	IEEE J Biomed Health Inform	USA	176	2,902	Q1	6.8	28

IF, Impact Factor, accessed from JCR 2025.

### 3.6 Author productivity and impact

**Figure 6:**
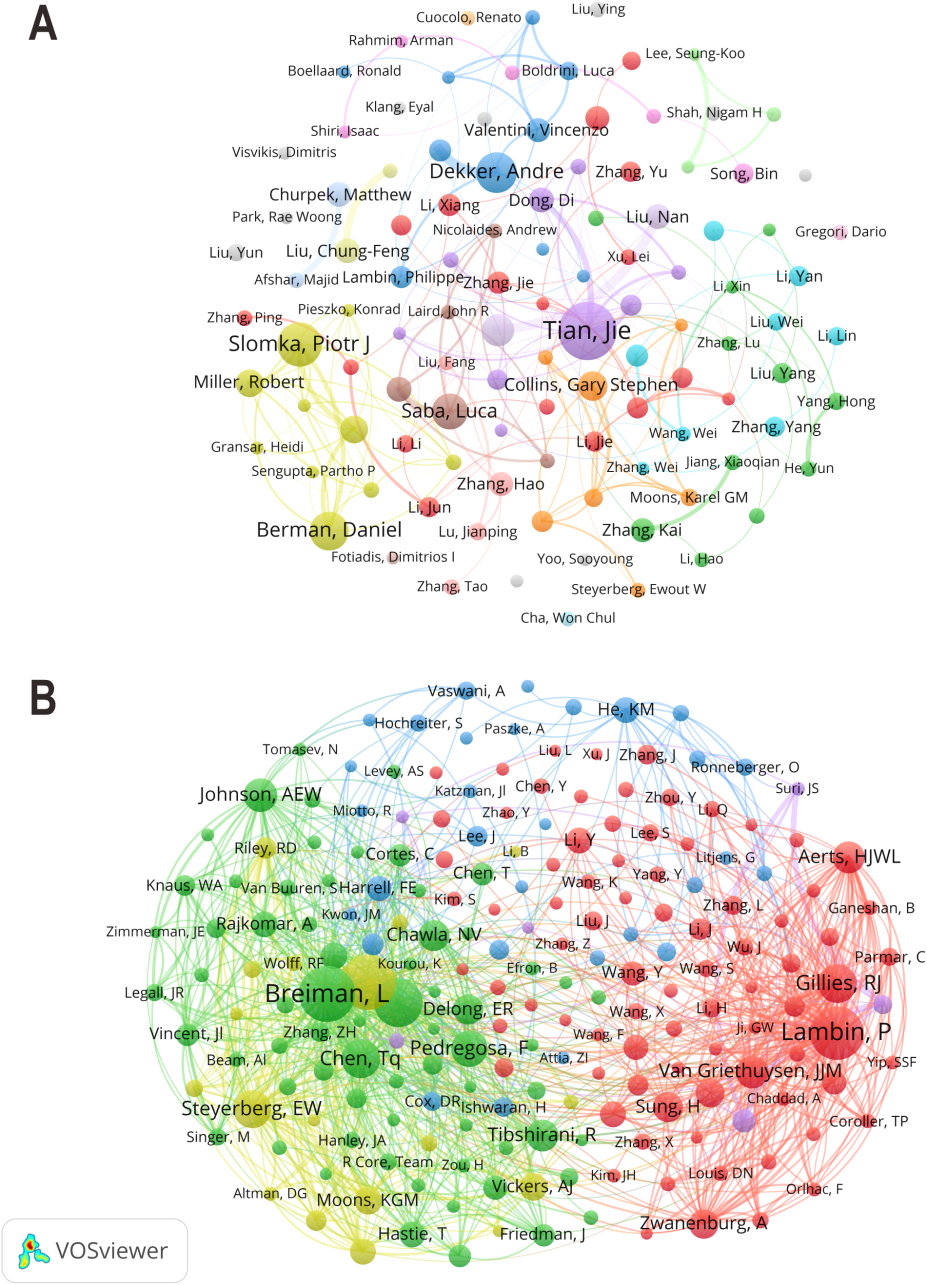
**(A)** Author collaboration network (authors with ≥10 publications). Each node represents an author, with node size indicating publication volume and line thickness reflecting the strength of collaboration. Colors denote different collaborative clusters. **(B)** Author co-citation network. Each node represents a cited author, with node size indicating citation frequency and connecting lines representing co-citation relationships between authors. Different colors indicate clusters of authors frequently cited together.

A total of 50,425 authors were identified across the 8,619 included publications. As shown in **Figure 6A**, the collaboration network of the top 100 most productive authors showed that Slomka PJ (TLS = 116), Berman DS (TLS = 105), Dey D (TLS = 105), and Tian J (TLS = 90) held central roles, indicating significant research activity and strong collaborative ties. In contrast, the author co-citation network in **Figure 6B** illustrated the scholarly influence of researchers through citation relationships. In this network, Collins GS (TLS = 6,287), Lambin P (TLS = 5,447), and Breiman L (TLS = 4,872) exhibited the highest co-citation strength, underscoring their substantial academic impact in the ML-CPMs domain. According to publication statistics in **Table 4**, Tian J from China ranked first with 42 papers and the highest H-index of 28. Slomka PJ from the United States and Dekker A from the Netherlands followed in second and third places, respectively. Notably, although Collins GS from the United Kingdom had a relatively modest publication count, his total citations (5,955) and average citations (270.68) were significantly higher than those of other authors, reflecting the broad recognition and high influence of his scientific contributions.

**Table 4:** Top 10 productive authors with publications.

Rank	Author	NP	NC	AC	H-Index	Country	Institution
1	Tian J	42	1,999	47.60	28	China	Chinese Academy of Sciences
2	Slomka PJ	33	1,295	39.24	17	USA	Cedars-Sinai Medical Center
3	Dekker A	30	1,200	40.00	17	Netherlands	Maastricht University
4	Berman DS	29	1,331	45.90	17	USA	Cedars-Sinai Medical Center
5	Dey D	29	1,141	39.34	16	USA	Cedars-Sinai Medical Center
6	Saba L	27	880	32.59	18	Italy	University of Cagliari
7	Ong MEH	24	620	25.83	13	Singapore	Singapore General Hospital
8	Collins GS	22	5,955	270.68	16	United Kingdom	University of Oxford
9	Miller RJH	21	401	19.1	12	Canada	University of Calgary
10	Churpek MM	19	778	40.95	11	USA	University of Wisconsin-Madison

NP, Number of Publications. NC, Number of Citations. AC, Average Citations. USA, the United States of America.

### 3.7 Cited and co-cited references

Regarding highly cited publications, the top ten papers identified in this study were primarily published in *BMJ, Nature Medicine, and NPJ Digital Medicine*, encompassing the fields of clinical medicine, epidemiology, and medical informatics (**Table 5**). Among these, the most frequently cited publication was the systematic review by Wynants L, published in *BMJ* in 2020, titled “Prediction models for diagnosis and prognosis of COVID-19 infection: systematic review and critical appraisal” (22). This study systematically evaluated diagnostic and prognostic prediction models related to COVID-19 and assessed their methodological quality, accumulating 2,049 citations to date.

**Table 5:** Top 10 highly cited publications.

Rank	Publications title	Journal	First author	Year	NC	Document Type
1	Prediction models for diagnosis and prognosis of covid-19infection: systematic review and critical appraisal	BMJ	Wynants L (22)	2020	2,005	Review
2	Diffuse large B-cell lymphoma outcome prediction by gene-expression profiling and supervised machine learning	Nat Med	Shipp MA (23)	2002	1,856	Article
3	Scalable and accurate deep learning with electronic health records	NPJ Digit Med	Rajkomar A (24)	2018	1,407	Article
4	Advantages and disadvantages of using artificial neural networks versus logistic regression for predicting medical outcome	J Clin Epidemiol	Tu JV (25)	1996	1,320	Review
5	A systematic review shows no performance benefit of machine learning over logistic regression for clinical prediction models	J Clin Epidemiol	Christodoulou E (26)	2019	1,119	Review
6	Calibration: the Achilles heel of predictive analytics	BMC Med	Van Calster B (27)	2019	980	Article
7	Comparing different supervised machine learning algorithms for disease prediction	BMC Med Inform Decis Mak	Uddin S (28)	2019	801	Article
8	A five-gene signature and clinical outcome in non-small-cell lung cancer	N Engl J Med	Chen HY (29)	2007	753	Article
9	A radiomics model from joint FDG-PET and MRI texture features for the prediction of lung metastases in soft-tissue sarcomas of the extremities	Phys Med Biol	Vallières M (30)	2015	704	Article
10	Machine learning in medicine: a practical introduction	BMC Med Res Methodol	Sidey-Gibbons JAM (31)	2019	685	Article

NC, Number of Citations.

**Figure 7:**
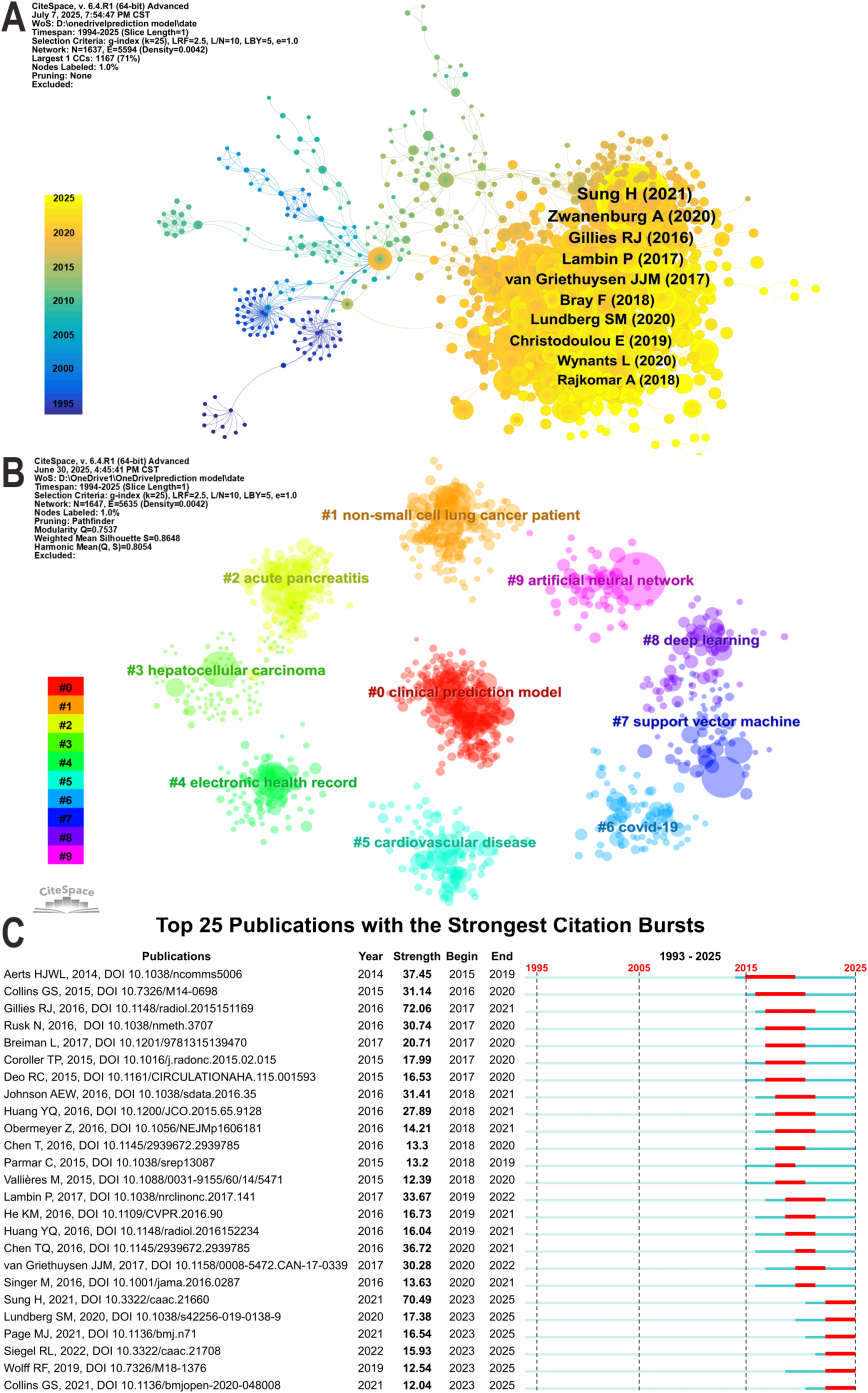
**(A)** Co-citation network of the 10 most-cited references. The g-index (k = 25) was used as the scaling factor, node size corresponds to co-citation frequency, and labels indicate the first author and publication year. **(B)** Clustering of cited references. Clusters are numbered based on reference keywords, smaller numbers denote larger clusters. **(C)** Top 25 references with the strongest citation bursts. Blue bars indicate the overall time span, and red segments mark the burst-active periods.

Based on 8,619 original publications, a total of 1,637 co-cited references were identified to construct the co-citation network (**Figure 7A**). The most frequently co-cited paper was “Global cancer statistics 2020: GLOBOCAN estimates of incidence and mortality worldwide for 36 cancers in 185 countries” (32), with 345 co-citations. This study provided a comprehensive assessment of the global cancer burden using data from GLOBOCAN 2020. Other highly co-cited papers among the top ten addressed topics such as radiomics standardization, explainable artificial intelligence (XAI), methodological comparisons of prediction models, and ML algorithms. Subsequent clustering analysis of co-cited references identified ten major thematic clusters (**Figure 7B**), with Modularity Q = 0.7537 and Weighted Mean Silhouette S = 0.8648. Both values exceeding 0.75 indicated that the clustering structure was well defined and internally consistent. The clusters covered topics including clinical prediction model development methods, applications of electronic health records, and disease-specific studies such as non-small cell lung cancer, acute pancreatitis, hepatocellular carcinoma, cardiovascular disease, and COVID-19. Moreover, **Figure 7C** presents the top 25 publications with the strongest citation bursts. The review by Gillies RJ, published in 2016, exhibited the highest burst intensity (Strength = 72.06) and remained active until 2021. This paper introduced the concept and research framework of radiomics and provided a detailed description of the complete workflow from image acquisition and feature extraction to model construction and clinical application (33). Seven publications showed ongoing citation bursts extending through 2025, most of which were published within the past five years and focused on cancer epidemiology, model interpretability, reporting standards for prediction models, and radiomics modeling.

### 3.8 Keyword co-occurrence and cluster analysis

**Figure 8:**
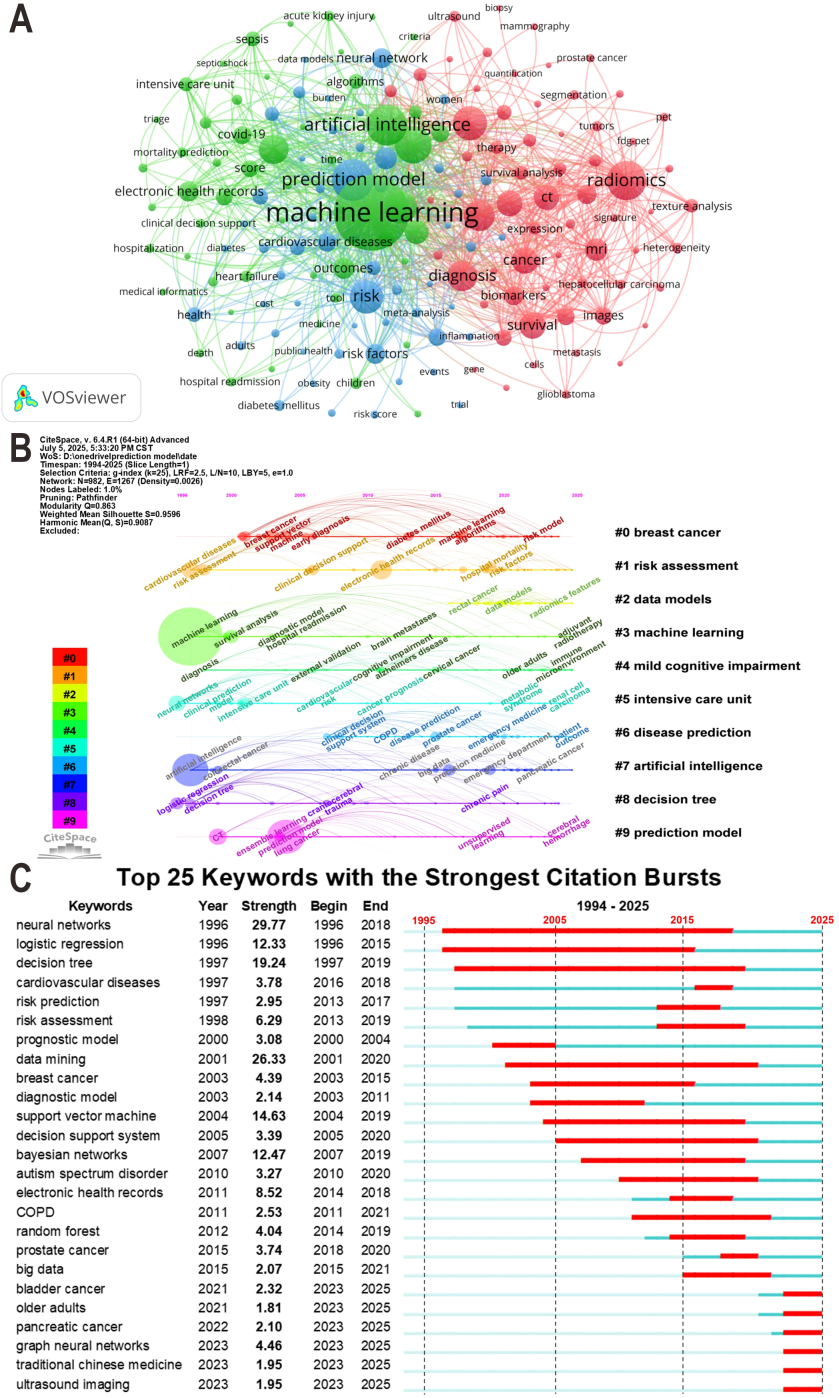
**(A)** Keyword co-occurrence network. This map highlights clusters of interrelated terms, each representing a distinct research domain. **(B)** Timeline view of keyword clusters. Horizontal lines indicate clusters, with labels displayed on the right side of the figure. Different colors denote separate clusters, and smaller cluster numbers correspond to larger cluster sizes. The position of nodes along the timeline reflects the temporal evolution of research topics. **(C)** Keyword burst analysis. Red line segments indicate burst periods, representing time intervals when the corresponding keywords received heightened research attention.

A total of 20,030 keywords were extracted from the analyzed publications. Based on their semantic features and contextual relevance within ML-CPM research, high-frequency keywords were grouped into five categories: technical methods, model development, disease applications, clinical settings, and clinical outcomes. **Table 6** lists the top three high-frequency keywords in each category. To further clarify the thematic landscape of ML-CPM research, we conducted a keyword co-occurrence analysis. As shown in **Figure 8A**, the co-occurrence network consisted of three clusters: medical imaging and oncology-related research (red cluster), AI methodologies and their application in hospital settings (green cluster), and prediction models and health outcomes (blue cluster). Overall, machine learning (TLS = 12,787) occupied a central position in the network, showing strong linkages with prediction model (TLS = 4,989), artificial intelligence (TLS = 4,934), and risk (TLS = 3,980).

**Table 6:** High-frequency keywords and their classifications.

Classification	Top 3	Count	TSL
Technical methods	machine learning	3,328	12,787
	artificial intelligence	1,199	4,934
	radiomics	1,133	4,940
Model development	prediction model	1,247	4,989
	risk	886	3,980
	classification	886	3,473
Disease applications	cancer	580	2,537
	cardiovascular diseases	326	1,443
	COVID-19	312	1,123
Clinical settings	electronic health records	363	1,611
	intensive care unit	214	1,078
	clinical decision support	99	476
Clinical outcomes	outcomes	405	1,877
	mortality prediction	144	539
	hospital readmission	120	538

TLS, Total Link Strength.

The keyword timeline view revealed the dynamic evolution of research themes within the ML-CPM field (**Figure 8B**), identifying ten major clusters with high structural clarity and internal consistency (Modularity Q = 0.863; Weighted Mean Silhouette S = 0.9596). Cluster #0 “breast cancer” primarily involved studies related to support vector machines and early diagnosis in breast cancer, whereas #3 “machine learning” focused on diagnostic modeling, prognostic analysis, and hospitalization risk assessment. In contrast, #7 “artificial intelligence” and #9 “prediction model” emerged primarily over the past five years, representing frontier hotspots in the field. **Figure 8C** summarizes the top 25 keywords with the strongest citation bursts. Early emerging hotspots included neural networks (Strength = 29.77), logistic regression (Strength = 12.33), and decision tree (Strength = 19.24), which were most active between 2000 and 2015. More recent bursts were observed for keywords such as graph neural networks, bladder cancer, traditional Chinese medicine, ultrasound imaging, and older adults, beginning in or after 2023 and indicating their growing prominence in the recent literature.

## 4 Discussion

Since Breiman L introduced the random forest algorithm in 2001, a solid methodological foundation has been established for the development of clinical prediction models (34). Research on prediction model construction based on machine learning has since gained widespread attention, with the number of related publications increasing exponentially. Early studies were predominantly conducted in the United States, the United Kingdom, and Germany, where well-established research infrastructures and access to large-scale medical databases fostered international collaboration and academic leadership. Over the past decade, China has rapidly emerged as a major contributor, driven by increased research investment, data-sharing initiatives, and supportive AI policies. This shift has given rise to a global pattern characterized by leadership from developed countries and rapid progress among developing nations. At the institutional level, Harvard University and the University of California System in the influence over time. Meanwhile, Chinese institutions such as Sun Yat-sen University, Fudan University, and Capital Medical University have demonstrated sustained growth in publication output, reflecting a notable rise in research activity. Collectively, these developments have fueled the global expansion of ML-CPMs research, closely linked to the rise of deep learning, the increasing availability of open-access medical databases, and the policy momentum toward precision medicine.

Journals, authors, and their representative publications collectively constitute the core network of knowledge dissemination and academic accumulation in this field, serving as key carriers of research productivity and scholarly influence. *BMC Medical Informatics* and *Decision Making* published the largest number of papers, consistently focusing on clinical data mining, decision support, and model validation, and has maintained high visibility within the domain of medical informatics. *Diagnostics* and *the Journal of Clinical Medicine* have also made notable contributions in radiomics, disease risk prediction, and model performance evaluation, indicating a growing trend toward the integration of ML-CPMs research with clinical practice. Among the leading researchers, Professor Tian J from the Chinese Academy of Sciences stands out as the most prolific contributor in this field, having authored 42 publications. In a 2022 article published in The Lancet Digital Health, his team demonstrated the clinical potential of deep learning for predicting chemoradiotherapy response in rectal cancer (35). In contrast, British scholar Collins GS is widely recognized for his methodological contributions, leading the development of the TRIPOD-AI reporting guideline that has greatly improved transparency and reproducibility in model development and validation (36). In parallel, several highly cited publications have further shaped the methodological foundations of ML-CPMs. Rajkomar A introduced deep learning frameworks for electronic health records (24), showcasing the power of machine learning to capture complex clinical patterns. Christodoulou E conducted a systematic review comparing the performance of machine learning and traditional statistical models (26), providing important insights into their relative advantages and limitations. Lambin P, through his pioneering research in radiomics (37), advanced the integration of imaging features into predictive modeling. Taken together, these studies have focused on algorithmic optimization, data integration, and multimodal modeling, reflecting a strong methodological and thematic alignment among leading contributors within the field.

By integrating the visualized results of keyword and co-citation analyses, research on clinical prediction models has shown a clear methodological evolution from conventional statistical approaches for risk prediction to intelligent model development driven by ML algorithms. Early investigations, characterized by keywords such as “logistic regression” and “decision tree,” primarily focused on variable selection, model calibration, and individualized risk prediction, emphasizing the feasibility and internal validation of traditional statistical methods (27, 38–40). With advances in computational capacity and the widespread adoption of electronic health records, keywords including “support vector machine,” “random forest,” and “data mining” appeared with increasing frequency, suggesting the increasing application of ML techniques in model development, comparison, and optimization. Notably, these terms were most prominent before 2019. Since then, research priorities have gradually shifted toward the construction and refinement of AI-driven CPMs. High-frequency keywords such as “machine learning,” “prediction model,” “artificial intelligence,” “radiomics,” and “deep learning” reflect the transition within the field toward multimodal feature integration and model performance enhancement. The combination of radiomics and deep learning represents one of the most active research frontiers, where multimodal feature extraction based on CT radiomics and 2.5D deep learning frameworks has demonstrated superior diagnostic accuracy in predicting tumor grade, recurrence, and metastasis risk (41–43).

Across major clinical disease domains, research on ML-CPMs has primarily concentrated on conditions with high prevalence and substantial disease burden, including chronic illnesses, acute and critical conditions, and infectious diseases. Among these, oncology has become the most mature and rapidly advancing research field, with studies mainly emphasizing cancer risk prediction, recurrence assessment, and treatment response modeling. The incorporation of deep learning has markedly improved model performance by enabling the automated extraction and representation of complex imaging and multimodal features, thereby driving the transition from algorithmic development toward precision medicine and individualized clinical decision-making (44–47). In comparison, cardiovascular research has placed greater emphasis on population-level risk stratification and long-term outcome prediction. ML-based models that combine imaging, pathologic, and physiologic data have demonstrated strong predictive stability and high accuracy across various clinical settings (48–50). In the field of infectious diseases, the COVID-19 pandemic has acted as a major catalyst for the rapid development of predictive modeling. The growing availability of large-scale clinical and imaging datasets has extended the application of ML-CPMs from single-disease risk prediction to more comprehensive assessments of overall health status (22). Furthermore, the application of ML-CPMs among critically ill and older adults presents unique challenges due to disease complexity, multiple comorbidities, and rapidly changing clinical conditions. These challenges have highlighted the need for improved model robustness, real-time adaptability, and interpretability. Consequently, XAI approaches have been increasingly adopted to enhance model transparency, improve interpretability, and promote greater confidence among clinicians in AI-assisted decision-making (51, 52).

Despite considerable advances in ML-CPMs, several unresolved challenges in the current evidence base continue to hinder clinical translation. A persistent imbalance remains between rapid model development and insufficient attention to comprehensive evaluation. Although discrimination is frequently reported, calibration, clinical utility, and performance heterogeneity across patient subgroups and care settings are less consistently examined, despite established methodological guidance emphasizing their importance for clinical decision-making (53, 54). External validation and assessment of transportability across institutions and health systems are also less common than model development, raising concerns about robustness and applicability in routine clinical practice (54). Reporting and appraisal frameworks have strengthened expectations for transparency, reproducibility, and risk-of-bias assessment, yet adherence remains variable (36, 55). Differences in data processing and predictor modeling across studies further limit comparability and hinder cumulative evidence synthesis. While explainable AI techniques aim to enhance interpretability, their reliability and clinical usefulness across diverse settings remain uncertain (56, 57). Addressing these limitations requires shifting research priorities from model construction alone toward rigorous external validation, transportability-oriented performance evaluation, and stronger adherence to reporting standards. Growing emphasis has also been placed on model lifecycle management, including post-deployment monitoring and systematic model updating to address distributional shift and maintain performance and reliability over time (58, 59).

The broader implementation of ML-CPMs increasingly depends on secure and privacy-preserving data infrastructures that enable cross-institutional collaboration while protecting patient confidentiality. Data sharing across institutions is frequently constrained by regulatory, legal, and organizational requirements (60). Even distributed learning approaches such as federated learning do not fully eliminate privacy risks, as sensitive information may still be inferred from model parameters or updates (61). Together, these constraints and residual privacy risks underscore the need for rigorous security design. This includes secure aggregation, differential privacy methods, and clearly defined governance frameworks to ensure accountability in data stewardship and model oversight (62). Current evidence suggests that large-scale clinical deployment of privacy-preserving machine learning remains limited relative to methodological development (63, 64). This highlights the need to evaluate privacy–utility trade-offs and security risks in addition to predictive performance.

This study presents several limitations. Our dataset was retrieved from WoSCC and Scopus, and relevant publications indexed exclusively in other databases may have been missed. Compared with prevalent bibliometric workflows that rely on a single database and a single mapping tool, our dual-database and multi-tool pipeline broadens coverage and enables cross-tool triangulation. The strengths of this approach include retrieval breadth and cross-tool validation. A key weakness is that citation-lag effects may underrepresent emerging topics, as recently published studies have had limited time to accumulate citations. Looking ahead, opportunities lie in pairing bibliometric mapping with systematic model evaluation, thereby linking identified hotspots to evidence quality and clinical relevance. Meanwhile, threats include evolving database indexing policies and software updates that may affect longitudinal comparability. Despite these limitations, this study offers a multidimensional overview of the development and research landscape of ML-CPMs over the past three decades, providing valuable insights for researchers in the field.

## 5 Conclusion

From 1994 to 2025, global research on ML-CPMs has shown sustained growth, reflecting a clear evolution from methodological innovation toward clinical implementation. Over time, this field has expanded to encompass diverse diseases and clinical applications. The United States continues to lead in academic influence and methodological standardization, whereas China has exhibited rapid growth in publication output, underscoring regional differences in research productivity and quality. Current research hotspots primarily focus on algorithmic optimization, integration of imaging and multi-omics data, model interpretability, and clinical validation. Overall, ML has played a pivotal role in advancing the development and optimization of CPMs, marking a transition from model development to a more clinically oriented phase of integrated innovation. As the field continues to evolve, with the increasing integration of multi-source data and the advancement of intelligent decision-support systems, ML-CPMs are expected to deliver broader and more sustainable clinical value in disease prediction, prognostic assessment, and precision intervention.

## Data Availability

The original contributions presented in the study are included in the article/**Supplementary Material**, further inquiries can be directed to the corresponding author/s.
