# Supplementary material for: Research on machine learning-based clinical prediction models: a bibliometric analysis
Source: Front Oncol. 2026 Apr 1;16:1786176. doi: 10.3389/fonc.2026.1786176 (PMC13078998; doi:10.3389/fonc.2026.1786176)
Supplement: Supplementary file 1 [file Table1.docx]

Supplementary Material

# Supplementary Table 1

| Supplementary Table 1. Search Strategy | | | |
| --- | --- | --- | --- |
| Database | Set | Search query | Results |
| WoSCC | #1 | TS = ("machine learning" OR "deep learning" OR "neural network*" OR "random forest*" OR "support vector machine*" OR "XGBoost" OR "supervised learning" OR "gradient boosting" OR "decision tree*" OR "ensemble model*" OR "multilayer perceptron*" OR "Bayes* network*" OR "artificial intelligen*" OR "large language model*" OR "natural language processing" OR "NLP" OR "transformer model*" OR "computer vision" OR "image recognition" OR "radiomics") | 1,800,972 |
|  | #2 | TS = ("clinical prediction model*" OR "prediction model*" OR "predictive model*" OR "diagnostic model*" OR "prognostic model*" OR "prediction tool*" OR "risk prediction" OR "mortality prediction" OR "readmission prediction" OR "survival prediction" OR "risk model*" OR "risk assess*" OR "risk stratification" OR "risk classif*" OR "risk score*" OR "risk scoring system" OR "risk scale*" OR "prognostic score*" OR "prognostic index") | 642,207 |
|  | #3 | #1 AND #2 | 116,448 |
|  | #4 | #3 AND TS = ("patient*" OR "clinic*" OR "medical" OR "hospital*" OR "healthcare" OR "disease*") | 40,350 |
|  | #5 | #4 AND SU = ("Medicine" OR "Medical Informatics" OR "Health Care Sciences Services") | 11,187 |
| Scopus | ( TITLE-ABS-KEY( "clinical prediction model*" OR "prognostic model*" OR "diagnostic model*" OR "machine learning model*" OR "AI-based model*" OR "ML-based model*" ) OR TITLE-ABS-KEY( ( "prediction model*" OR "predictive model*" OR "risk model*" ) W/5 ( "machine learning" OR "deep learning" OR "artificial intelligence" OR "AI" ) ) ) AND TITLE-ABS-KEY( "machine learning" OR "deep learning" OR "neural network*" OR "random forest*" OR "support vector machine*" OR "XGBoost" OR "gradient boosting" OR "decision tree*" OR "artificial intelligen*" OR "radiomics" ) AND TITLE-ABS-KEY( patient* OR clinical* OR hospital* OR healthcare* ) AND SUBJAREA( MEDI ) AND SRCTYPE( j ) AND ( DOCTYPE( ar ) OR DOCTYPE( re ) ) AND LANGUAGE( english ) | | 13,403 |
