# Supplementary material for: Research on machine learning-based clinical prediction models: a bibliometric analysis
Source: Front Oncol. 2026 Apr 1;16:1786176. doi: 10.3389/fonc.2026.1786176 (PMC13078998; doi:10.3389/fonc.2026.1786176)
Supplement: Supplementary file 2 [file Table2.docx]

Supplementary Material

# Supplementary Table 2

| Supplementary Table 2. Top 10 highly co-cited publications | | | | | | |
| --- | --- | --- | --- | --- | --- | --- |
| Rank | Publications title | Journal | First author | Year | Co-citation | Document Type |
| 1 | Global cancer statistics 2020: GLOBOCAN estimates of incidence and mortality worldwide for 36 cancers in 185 countries | CA-CANCER J CLIN | Sung H | 2021 | 345 | Article |
| 2 | The Image Biomarker Standardization Initiative: Standardized Quantitative Radiomics for High-Throughput Image-based Phenotyping | RADIOLOGY | Zwanenburg A | 2020 | 245 | Article |
| 3 | Radiomics: Images Are More than Pictures, They Are Data | RADIOLOGY | Gillies RJ | 2016 | 232 | Article |
| 4 | Radiomics: the bridge between medical imaging and personalized medicine | NAT REV CLIN ONCOL | Lambin P | 2017 | 200 | Review |
| 5 | Computational Radiomics System to Decode the Radiographic Phenotype | CANCER RES | van Griethuysen JJM | 2017 | 184 | Article |
| 6 | Global cancer statistics 2018: GLOBOCAN estimates of incidence and mortality worldwide for 36 cancers in 185 countries | CA-CANCER J CLIN | Bray F | 2018 | 167 | Article |
| 7 | From local explanations to global understanding with explainable AI for trees | NAT MACH INTELL | Lundberg SM | 2020 | 154 | Article |
| 8 | A systematic review shows no performance benefit of machine learning over logistic regression for clinical prediction models | J CLIN EPIDEMIOL | Christodoulou E | 2019 | 135 | Review |
| 9 | Prediction models for diagnosis and prognosis of covid-19 infection: systematic review and critical appraisal | BMJ-BRIT MED J | Wynants L | 2020 | 121 | Review |
| 10 | Scalable and accurate deep learning with electronic health records | NPJ DIGIT MED | Rajkomar A | 2018 | 108 | Article |
